# Effects of long-term elevated temperature on covering, sheltering and righting behaviors of the sea urchin *Strongylocentrotus intermedius*

**DOI:** 10.7717/peerj.3122

**Published:** 2017-03-22

**Authors:** Lisheng Zhang, Lingling Zhang, Dongtao Shi, Jing Wei, Yaqing Chang, Chong Zhao

**Affiliations:** Key Laboratory of Mariculture & Stock Enhancement in North China’s Sea, Ministry of Agriculture, Dalian Ocean University, Dalian, China

**Keywords:** Sea urchin, *Strongylocentrotus intermedius*, Ocean warming, Behavior

## Abstract

Increases in ocean temperature due to climate change are predicted to change the behaviors of marine invertebrates. Altered behaviors of keystone ecosystem engineers such as echinoderms will have consequences for the fitness of individuals, which are expected to flow on to the local ecosystem. Relatively few studies have investigated the behavioral responses of echinoderms to long-term elevated temperature. We investigated the effects of exposure to long-term (∼31 weeks) elevated temperature (∼3 °C above the ambient water temperature) on covering, sheltering and righting behaviors of the sea urchin *Strongylocentrotus intermedius*. Long-term elevated temperature showed different effects on the three behaviors. It significantly decreased covering behavior, including both covering behavior reaction (time to first covering) and ability (number of covered sea urchins and number of shells used for covering). Conversely, exposure to long-term elevated temperature significantly increased sheltering behavior. Righting response in *S. intermedius* was not significantly different between temperature treatments. The results provide new information into behavioral responses of echinoderms to ocean warming.

## Introduction

The Intergovernmental Panel on Climate Change (IPCC) predicts that global mean sea water temperature will increase 2–4.5 °C by 2100 (scenario RCP 8.5, IPCC, 2013). Consistent with theoretical predictions (Poloczanska et al., 2007), empirical studies indicate that elevated water temperature negatively affects physiology (Uthicke et al., 2014), immunity (Brothers et al., 2016), growth (Wolfe, Dworjanyn & Byrne, 2013; Zhao et al., 2015), gonad development (Uthicke et al., 2014), larval development (Kamya et al., 2014; Garcia, Clemente & Hernandez, 2015) and behaviors (Zhao et al., 2014; Brothers & McClintock, 2015; Sherman, 2015) of echinoderms in intertidal and shallow waters. However, most of these reports were based on short-term perturbation experiments and do not provide enough time to bring about potential acclimation to a new environment (Dupont et al., 2013). For example, 8–20 weeks exposure to a substrate with cavities significantly reshaped the test height:diameter of the sea urchin *Strongylocentrotus purpuratus*, while short-term exposure did not (Hernández & Russell, 2010). Long exposure time studies can provide much more valuable information, although the experimental durations (several months) are still relatively short in terms of their life span (Dupont et al., 2013).

The sea urchin *Strongylocentrotus intermedius* is an ecologically important species both as an herbivorous grazer of *Saccharina* spp. and other macroalgal species (for example, *Ulva lactuca*) (Fuji, 1967), and as prey for crabs and starfish (Agatsuma, 2013). The species is distributed throughout the cold waters of Hokkaido, Japan as well as Korea, Far East Russia and China in intertidal and shallow waters no more than 13 m deep (Agatsuma, 2013). The upper lethal temperature limit is ∼23 °C for juveniles (Hokkaido Central Fisheries Experimental Station, Shiribeshihokubu Fisheries Extension Office & Hokkaido Institute of Mariculture , 1984). Echinoderm behaviors are important for their fitness, but also affect the marine ecosystem through effects on their prey, predators and competitors (Ling & Johnson, 2012). Like other echinoids, *S. intermedius* has a number of discrete behaviors, including covering, sheltering and righting behaviors. Covering behavior refers to sea urchins using their tube feet and spines to move objects, such as shells, stones and algal fragments, onto their dorsal surface (Verling, Crook & Barnes, 2002; Pawson & Pawson, 2013). Whereas sheltering behavior is a common habit of sea urchins to inhabit shelters (from small crevices to large reefs). Righting is the behavior of an inverted sea urchin to resume the posture with the aboral side up (Hyman, 1955). All three behaviors are ecologically important. Covering and sheltering behaviors provide protection against solar radiation (Dumont et al., 2007; Pinna et al., 2012) and against predation (Agatsuma, 2001). The righting response, on the other hand, is very important for sea urchins to escape from predators and physical turbulence (Brothers & McClintock, 2015). As a representative cold water species, *S. intermedius* is a good research model to investigate long-term effects of elevated temperature on behavioral responses of an important ecological engineer.

The main aim of the present study is to investigate the effects of long-term elevated temperature on covering, sheltering and righting behaviors of *S. intermedius* in order to provide new information into behavioral responses of echinoderms as ecosystem engineers to ocean warming.

## Methods

### Sea urchins

Three thousand juvenile sea urchins were transported from Dalian Haibao Fishery Company (121°22′E 38°77′N) to the Key Laboratory of Mariculture and Stock Enhancement in North China’s Sea, Ministry of Agriculture at Dalian Ocean University (121°37′E 38°87′N) on March 5, 2015. The sea urchins were produced by methods of mixed breeding (mixing sperm and eggs of hundreds of parents) in October 2014. The gametes were from the second generation offspring of the introduced Japanese sea urchins from the farming area of the company. Common hatchery methods (Chang et al., 2012) were used to culture the larvae and juveniles. Because the two generations of ancestors of the sea urchins used in this study inhabit wild environments, the sampling method used would not influence experimental results and interpretation of ecologically applications.

One thousand juveniles were maintained in each of the three tanks (length × width × height: 850 × 500 × 650 mm, ∼276 L) at laboratory temperature and under natural photoperiod and fed freshly collected *Saccharina japonica* or *Ulva lactuca* until the start of the experiment. One third of the water was changed every morning.

### Temperature acclimation

**Figure 1:**
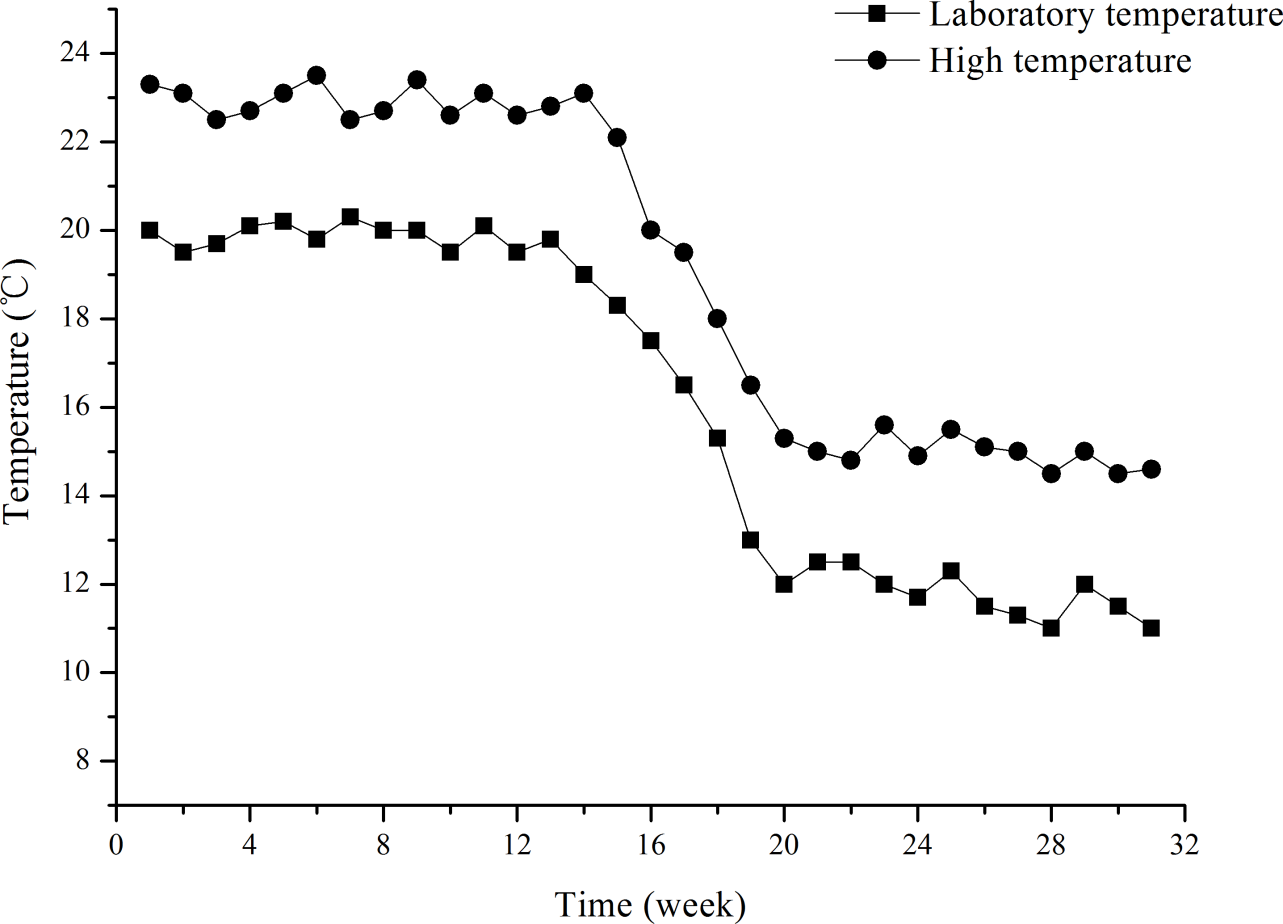
Water temperatures of groups L (laboratory temperature) and H (high temperature) during the experiment from July 8, 2015 to February 1, 2016. The designation was mainly based on the temperature of laboratory water, given the upper limits of 20 ± 0.5 °C and 23 ± 0.5 °C for groups L and H, respectively.

The water temperature treatments lasted for ∼31 weeks (from July 8, 2015 to February 1, 2016). The two water temperature treatments were laboratory temperature (group L) and high temperature (group H). Group L temperature was that of filtered, oxygenated and recirculated (0.12–0.15 L S^−1^) seawater pumped from the field. The temperature was measured daily. The study was a long-term exposure experiment, in which laboratory temperature reflects seasonal variation in temperature (Fig. 1). The temperature of group H was that of the same filtered, oxygenated and recirculated seawater increased to ∼3 °C higher than the group L temperature. Group H temperature was gradually increased from 20 °C (temperature of laboratory water at the beginning of the experiment) by 0.5 °C d^−1^ to 23 ± 0.5 °C (∼3 °C higher than the laboratory water). This temperature was then kept 3 °C higher than the Group L temperature throughout the experiment (∼31 weeks). Water temperatures of both groups L and H were continually regulated using a seawater temperature control system (Huixin Co., Foshan, Guangdong, China). In order to avoid mass mortality of juveniles, 23 ± 0.5 °C was set as the upper limit for group H, and consequently 20 ± 0.5 °C for group L, although field water temperatures were more than 20 °C in a number of days in summer. The water temperatures for groups L and H are shown in Fig. 1. Water temperature decreased from ∼20 °C to ∼11 °C in group L during the experiment.

At the beginning of the experiment, fifty juvenile *S. intermedius* were haphazardly collected from the initial group of sea urchins and distributed into each of three cages made of a plastic screen (200 × 300 × 400 mm, mesh size: 8 mm) inside three separate tanks (750 × 430 × 430 mm, ∼139 L). The screen allowed free water exchange in each tank. The three separate tanks in each temperature treatment shared the recirculated water. The area of the cages was sufficiently large to avoid interactions between urchins.

Test diameter and body weight of sea urchins were measured using a digital vernier caliper (Mahr Co., Göttingen, Germany) and an electric balance (G&G Co., Omaha, NE, USA). There was no significant difference in body weight of sea urchins between the experimental groups at the beginning of the experiment (1.67 ± 0.31 g, *P* > 0.05). In order to avoid potential damage, test diameter of only 10 randomly chosen sea urchins was measured in each group, showing no significant difference (10.0 ± 0.3 mm, *P* > 0.05). *Saccharina japonica* or *U. lactuca* were fed *ad libitum* during the experiment according to availability. Uneaten food and feces were removed. One third of the water was in each tank was changed every five days, in accordance with the recirculation and purification devices in the recirculated water system. The seawater salinity, pH and dissolving oxygen amount were measured irregularly during the experiment using a portable water quality monitoring meter (YSI, USA). They were 31.62–32.32‰, 7.97–8.25 and 5.1–6.6 g mL^−1^, respectively. This indicates that water quality was in the optimal ranges for *S. intermedius*, although the ranges were not trivial. There was no obvious difference in the periodicity between water changes.

At the end of the temperature acclimation, behavioral experiments of adults were carried out at both temperatures (11 °C and 14.6 °C) for groups L and H, from January 30 to February 1, 2016. Test diameter, height and body weight of sea urchins were measured after the behavioral experiments.

### Covering behavior

Covering behavior of 60 haphazardly chosen sea urchins from each treatment was tested in a plastic tank (500 × 350 × 300 mm) in a temperature controlled aquaria for each temperature treatment (*N* = 60). The tanks were sufficiently large to avoid interaction among the sea urchins. To avoid potential effects of biological rhythm on covering behavior (Zhao et al., 2013), the experiments were carried out at the same period of time among days for the two treatments. Two hundred shells of juvenile scallops *Patinopecten yessoensis* (20.0 ± 0.1 mm in shell height) were evenly spaced on the bottom of the testing tank as covering material. Time to first covering is the average time of covering by the first five *S. intermedius* that covered. The number of covered sea urchins and number of shells used by the sea urchin for covering were recorded 2 h after the beginning of the experiment.

### Sheltering behavior

**Figure 2:**
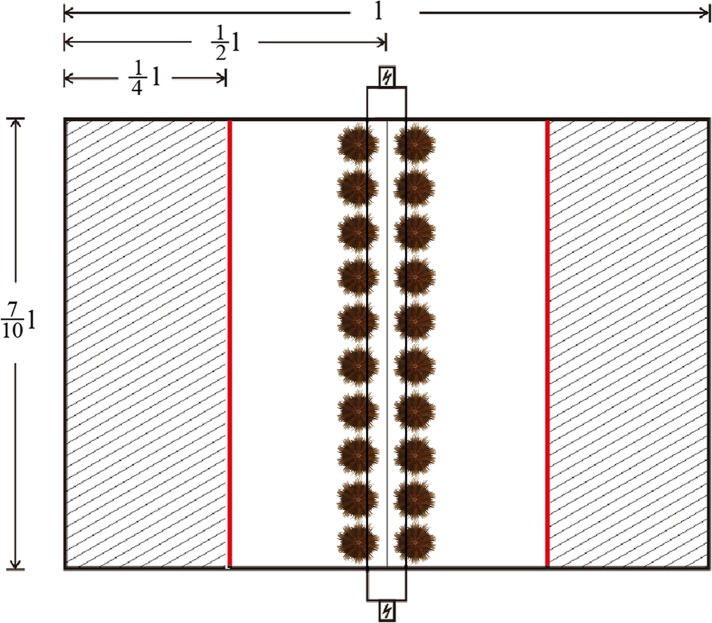
Diagram of the system used for testing sheltering behavior of *Strongylocentrotus intermedius*. The red line refers to the boundary lines between the lighted area and shaded areas. The letter “l” refers to the length of the tank.

Sheltering behavior of twenty sea urchins was tested in a rectangular plastic tank (500 × 350 × 300 mm) in temperature controlled aquaria of each experimental group. We repeated the experiments four times for both treatments, using different sea urchins each time (*N* = 4). The method was as follows:

One quarter of the area of the testing tank was shaded by opaque plastic sheets on both sides, with a fluorescent lamp (28W, Opple Co. China) 500 mm above the tank for illumination. The intensity of illumination was ∼120 lx in the lighted area and 5–10 lx in the shaded areas (Fig. 2). Twenty sea urchins were placed in the middle line of the testing tank before turning on the fluorescent lamp. Sheltering behavior was recorded when the sea urchin passed through the boundary lines between the lighted area and the shaded areas (the red lines in Fig. 2). The number of sheltered sea urchins was measured after 1, 2, 3, 4, 5, 6, 7, 8, 9, 10, 15, 20, 25 and 30 min.

### Righting response

Righting response of forty sea urchins was tested in a tank (600  × 350 × 200 mm) in the temperature controlled aquaria of each experimental group. The tanks were sufficiently large to avoid interaction among the sea urchins. Sea urchins were placed on the bottom of the tank with their oral side up. Righting response time was the length of time required for sea urchins to right themselves with aboral side up within 10 min.

### Statistical analysis

The data were tested for homogeneity of variance and normal distribution before the following statistical analyses. Independent-sample *t*-test was used to compare the differences of covering and righting behaviors between experimental groups. Time of sheltering response was analyzed using one-way repeated measured ANOVA. All data analysis was performed using SPSS 16.0 statistical software. A probability level of *P* < 0.05 was considered as significant.

## Results

### Test diameter and body weight

The sea urchins had grown to an average of ∼30 mm in test diameter and ∼12 g body weight by the end of the experiment. There was no significant difference in test diameter and body weight of sea urchins in the different water temperature treatments (*P* > 0.05).

### Covering behavior

**Figure 3:**
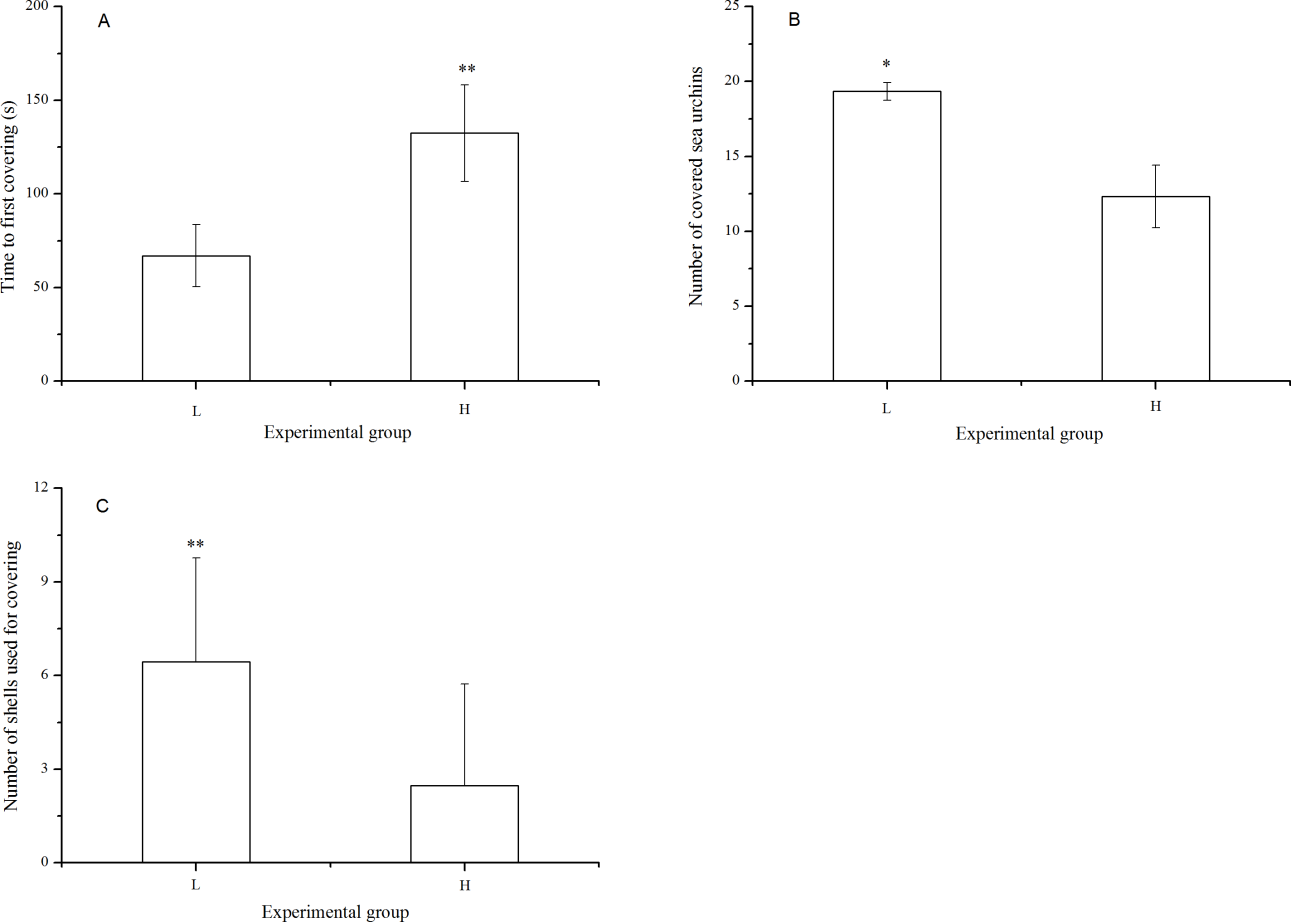
Time to first covering (A), number of covered sea urchins (B) and number of shells used for covering (C) of *Strongylocentrotus intermedius* in different experimental groups (mean ± SD). Significant differences are marked as * and ** for *P* < 0.01 and *P* < 0.001, respectively. L and H refer to laboratory and high temperature group, respectively.

There were significantly fewer covered sea urchins (*P* = 0.005) with significantly fewer shells used for covering (*P* < 0.001) when they were exposed to long-term high temperature. Consistently, the time to first covering was significantly lower in the sea urchins at the laboratory temperature (*P* < 0.001, Fig. 3).

### Sheltering behavior

**Figure 4:**
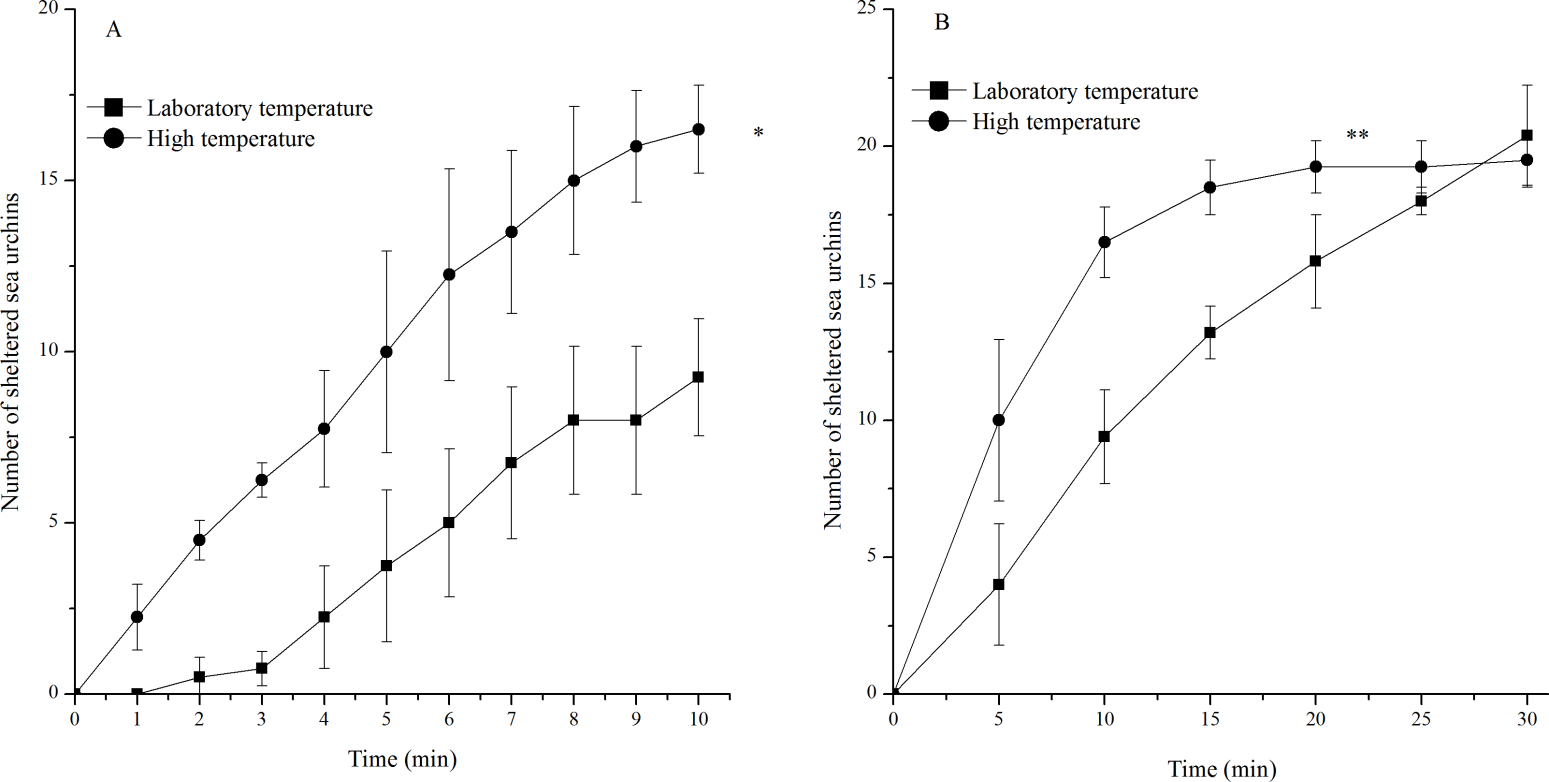
Number of sheltered *Strongylocentrotus intermedius* in the different experimental groups during 10 min (A) and 30 min (B) after the beginning of observations (mean ± SD). Significant differences were marked as * and ** for *P* < 0.01 and *P* < 0.001, respectively.

Sea urchins exposed to high temperature showed significantly more sheltering behavior during 10 min (*P* = 0.001) and 30 min (*P* < 0.001, Fig. 4). An average of 16 of the 20 sea urchins tested had sheltered at the high temperature, while only an average of nine of the 20 individuals tested had sheltered in the laboratory temperature group 10 min after the beginning of observations. However, the number of sheltered sea urchins in the two groups 30 min after the beginning of observations was similar (Fig. 4).

### Righting behavior

**Figure 5:**
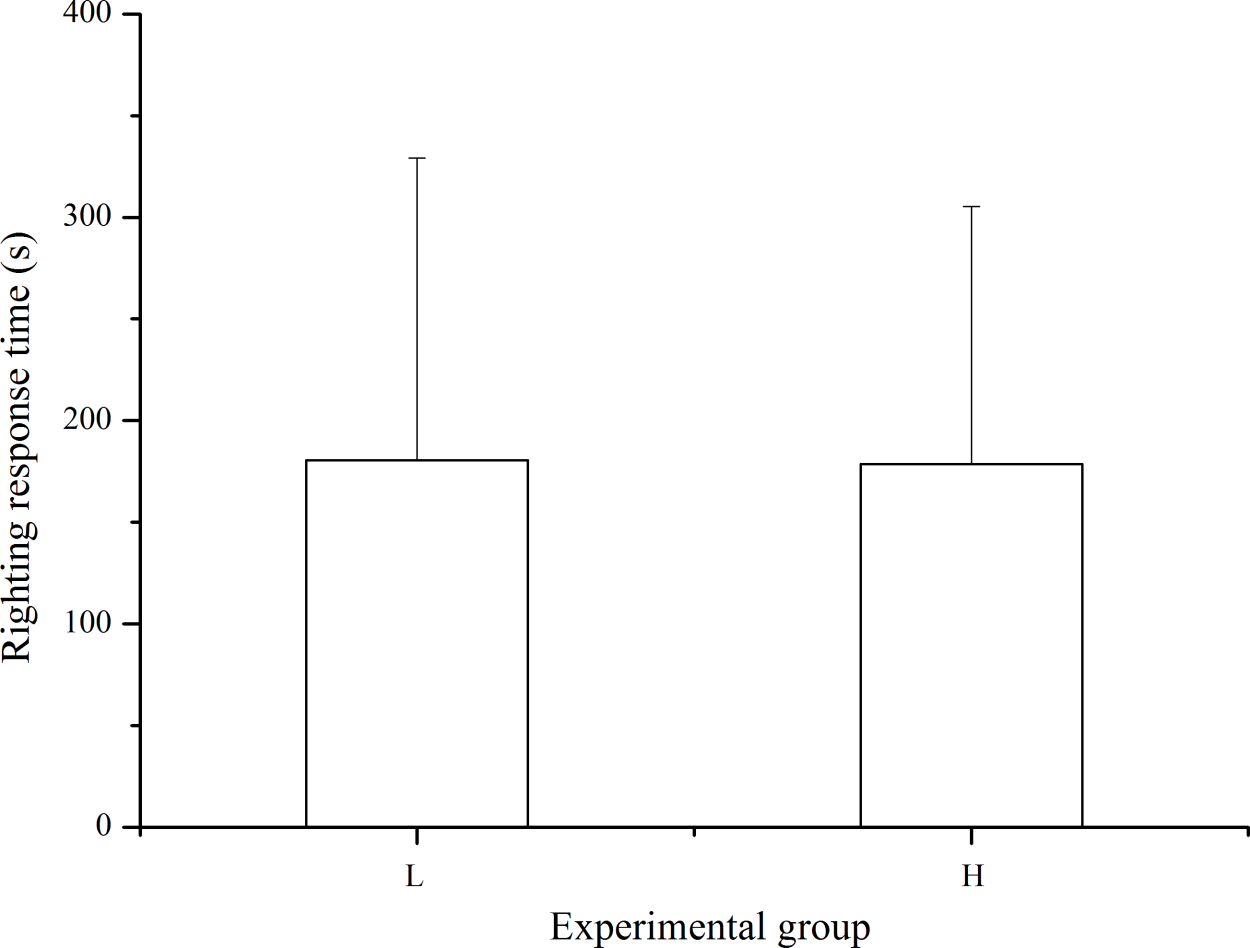
Righting time of *Strongylocentrotus intermedius* in the different experimental groups (mean ± SD). *N* = 33 for group L and *N* = 37 for group H. L and H refer to laboratory and high temperature groups, respectively.

Righting response was observed in 33 of the 40 sea urchins in the laboratory temperature group and in 37 of the 40 individuals in the high temperature group within 10 min after inverting the sea urchin. The differences in righting response was not significant between treatments (*P* > 0.05, Fig. 5).

## Discussion

Behavioral response of sea urchins is an important concern in climate change biology, because of their susceptibility and ecological importance in the marine ecosystems (Brothers & McClintock, 2015). Previous studies investigated the effects of short-term exposure to high temperature on the behaviors of sea urchins (Zhao et al., 2014; Brothers & McClintock, 2015; Sherman, 2015). However, longer term studies that allow for acclimation, more closely simulate the conditions of observed and expected ocean warming. The present study provides interesting and valuable information on the effects of long-term (∼31 weeks) elevated temperature (+∼3 °C) on covering, sheltering and righting behaviors of sea urchins. All experiments were performed with adult sea urchins, which grew up from juveniles during the experiment (∼31 weeks). Responses of juveniles to stress can be obviously different from those of adults.

Our previous study with short-term exposure found that there was significantly more covered *S. intermedius* at 15 °C than at 25 °C, but not significantly more than those at 5 °C (Zhao et al., 2014). The change in covering behavior at 15 °C and 25 °C may be explained by approaching the optimal and upper thermal tolerances of *S. intermedius* (Chang et al., 2004). The present study showed the same trend, with covering behavior significantly decreased by long-term elevated temperature even though the temperature at the time of the experiment was ∼15 °C. Both covering behavior reaction (time to first covering) and ability (number of covered sea urchins and number of shells used for covering) significantly decreased in the high temperature treatment indicating that the effect of long-term exposure to elevated temperature affects behavior in *S. intermedius* even when exposed to optimal temperatures. However, species and experimental conditions must also be considered as Brothers & McClintock (2015) found that *L. variegatus* exposed to 32 °C over ten days covered themselves less than those acutely exposed to the same temperature. Covering behavior has biological functions of protection against wave surge and floating debris (Millott, 1975; Richner & Milinski, 2000), predation (Agatsuma, 2001), and over-exposure to light (Verling, Crook & Barnes, 2002; Dumont et al., 2007). Thus, it is reasonable to infer that long-term elevated temperature impacts biological functions of sea urchins, consequently reducing fitness through the reduced covering behavior.

The present study found that the numbers of both quick (within 10 min) and moderate (within 30 min) sheltering responses of *S. intermedius* exposed to long-term elevated temperature were both significantly more than those that did not experience elevated temperature. The quick sheltering response is consistent with the known sheltering behavior of aestivating sea cucumbers *Apostichopus japonicus* at high temperature (Ji, Dong & Dong, 2008). Sheltering behavior has been suggested to attract sea urchins towards the edge of seagrass beds (Pinna, Sechi & Ceccherelli, 2013) and to prevent predation and over-exposure to light radiation (Farina et al., 2009). Thus, we hypothesize that increased sheltering response will have important consequences for sea urchin fitness from ocean warming. This hypothesis is supported by a recent finding that shelter was important for increased survival of the sea urchin *Centrostephanus rodgersii* with the rapidly warming Tasmanian east coast (Ling & Johnson, 2012). Kelp, as both food and shelter for a number of organisms including sea urchins, are particularly vulnerable to ocean warming due to their cold-water habitats and limited dispersal ability (Merzouk & Johnson, 2011), probably putting further pressures on sea urchin populations at higher latitudes. In the present study, the response of sheltering behavior of *S. intermedius* to elevated temperature was different from that of covering behavior. This supports our previous hypothesis that covering and sheltering behaviors probably do not share the same internal mechanism (Zhao, Bao & Chang, 2016).

Righting behavior is important for sea urchins to escape from adverse conditions (for example, dislocation by waves) to increase their survival in exposure to predators (Percy, 1973). Lawrence (1975) reported that *L. variegatus* exposed to 28 °C showed significantly higher righting ability than those exposed to 34 °C. Short-term exposure to 32 °C (from 25 °C to 32 °C for one week, and maintained at 32 °C for ten days) also had a significantly negative impact on righting behavior of *L. variegatus* (Brothers & McClintock, 2015). In the present study, however, exposure to long-term elevated temperature (+∼3 °C) did not significantly affect righting response time of *S. intermedius*, compared with those at the laboratory temperature. We suggest that long term exposure to higher temperatures allowed time for righting behaviors to acclimate to the conditions. However, species difference cannot be ignored as an alternative possibility.

In conclusion, exposure to long-term elevated temperature (∼31 weeks) showed different effects on covering, sheltering and righting behaviors of *S. intermedius*. Long-term high temperature significantly decreased covering behavior, including both covering behavior reaction (time to first covering) and ability (number of covered sea urchins and number of shells used for covering). However, sheltering behavior significantly increased in *S. intermedius* acclimated to elevated temperature. The righting response, on the other hand, showed no significant difference between *S. intermedius* acclimated to elevated temperature and the laboratory temperature. The present study provides new information into behavioral responses of echinoderms to ocean warming.

##  Supplemental Information

10.7717/peerj.3122/supp-1 Data S1 Raw data of behaviors and body size

10.7717/peerj.3122/supp-2 Supplemental Information 1 Water temperature setting during the experiment

## Additional Information and Declarations

### Data Availability

The following information was supplied regarding data availability: The raw data has been supplied as a Supplementary File.
